# Toxic retrobulbar neuritis due to recurrent nonsteroidal antiinflammatory drug-exacerbated respiratory disease-based chronic sinusitis in the left sphenoid sinus: a case report

**DOI:** 10.1186/s13256-023-04060-3

**Published:** 2023-08-04

**Authors:** Mirco Schapher, Jacob Bruegel, Fabian Guener, Bastian Volbers, Philip Eichhorn, Abbas Agaimy, Magdalena Berger, Christian Mardin, Arnd Doerfler, Stefan W. Hock

**Affiliations:** 1 Department of Otorhinolaryngology–Head and Neck Surgery, Erlangen University Hospital, University of Erlangen–Nuremberg, Waldstraße 1, 91054 Erlangen, Germany; 2 Department of Neurology, Erlangen University Hospital, University of Erlangen–Nuremberg, Schwabachanlage 6, 91054 Erlangen, Germany; 3 Department of Pathology, Erlangen University Hospital, University of Erlangen–Nuremberg, Krankenhausstraße 8-10, 91054 Erlangen, Germany; 4 Department of Ophthalmology, Erlangen University Hospital, University of Erlangen–Nuremberg, Schwabachanlage 6, 91054 Erlangen, Germany; 5 Department of Neuroradiology, Erlangen University Hospital, University of Erlangen–Nuremberg, Schwabachanlage 6, 91054 Erlangen, Germany

**Keywords:** Case report, Sinusitis, Blindness, Inflammation mediators, Optic, Neuritis

## Abstract

**Background:**

Abrupt visual impairment constitutes a medical urgency, necessitating an interdisciplinary diagnostic and therapeutic approach owing to the broad spectrum of potential etiologies, thereby engaging numerous medical specialties.

**Case presentation:**

A 21-year-old Mixed White and Asian female patient, with medical history of nonsteroidal antiinflammatory drug-exacerbated respiratory disease necessitating previous sinus surgery, reported sudden monocular vision loss. Unremarkable ophthalmological examination of the fellow eye and hematological parameters, save for a slight elevation in lymphocytes and eosinophils, were observed. Imaging studies indicated recurrence of bilateral chronic rhinosinusitis with nasal polyps and a mucocele in the left sphenoid sinus, accompanied by bony structural deficits. Emergency revision sinus surgery, guided by navigation, was promptly performed. The patient received treatment with methylprednisolone, ceftriaxone, cyanocobalamin, pyridoxine, thiamine, and acetylsalicylic acid. During the hospital stay, she developed steroid-induced glaucoma, which was subsequently managed successfully. Negative microbiological swabs, along with pathohistological evidence of increased tissue eosinophilia and the patient’s clinical history, led to the diagnosis of toxic retrobulbar neuritis secondary to recurrent nonsteroidal antiinflammatory drug-exacerbated respiratory disease-associated chronic rhinosinusitis of the left sphenoid sinus.

**Conclusions:**

In cases of acute unilateral vision loss, optic neuritis is a highly probable differential diagnosis and may be induced by pathologies of the paranasal sinuses. Nonsteroidal antiinflammatory drug-exacerbated respiratory disease, a subtype of chronic rhinosinusitis, is associated with type 2 inflammation, which is increasingly recognized for its role in the pathogenesis of bronchial asthma, eosinophilic esophagitis, and atopic eczema. Clinicians should consider chronic rhinosinusitis as a potential differential diagnosis in unilateral visual loss and be cognizant of the rising significance of type 2 inflammations, which are relevant to a variety of diseases.

## Background

Optic neuritis, a primary differential diagnosis for acute monocular vision loss, bifurcates into two subtypes [1]: typical optic neuritis, which usually presents unilaterally, either in isolation or in association with multiple sclerosis, and atypical optic neuritis, which is secondary to underlying pathologies (e.g., autoimmune diseases, post-vaccination reactions, parainfectious/postinfectious states), tumor infiltration, metabolic insufficiencies, toxic injuries, or neuronal ischemia, and can present as a unilateral or bilateral phenomenon.

Chronic rhinosinusitis (CRS), a potential etiology of atypical optic neuritis, is an inflammatory condition of the nasal passages and paranasal sinuses, with a prevalence of 4.5–12% in North America and Europe. Manifesting through persistent symptoms such as nasal congestion, rhinorrhea, olfactory dysfunction, facial pain, or headache for over 12 weeks, CRS is further classified by the presence or absence of nasal polyposis [2]. The pathogenesis of nasal polyps remains partially elucidated, but histopathological examinations suggest a dominant T-helper-type 2-cell (Th2) inflammatory response. This type 2 inflammation is implicated in the pathogenesis of various conditions, including bronchial asthma, eosinophilic esophagitis, or atopic eczema, thereby garnering increasing attention from multiple specialties and expanding treatment possibilities in recent years.

## Case presentation

A 21-year-old Mixed White and Asian woman reported sudden vision loss in her left eye. Initial examination revealed hand motion recognition in the affected eye, slight increase in lymphocytes and eosinophils, and normal vital signs. The patient’s medical history included mild spastic tetraparesis, treated hypothyroidism, and nonsteroidal anti-inflammatory drug (NSAID)-exacerbated respiratory disease (NERD) with associated chronic rhinosinusitis, asthma, and cyclooxygenase (COX)-1 inhibitor intolerance.

**Fig. 1:**
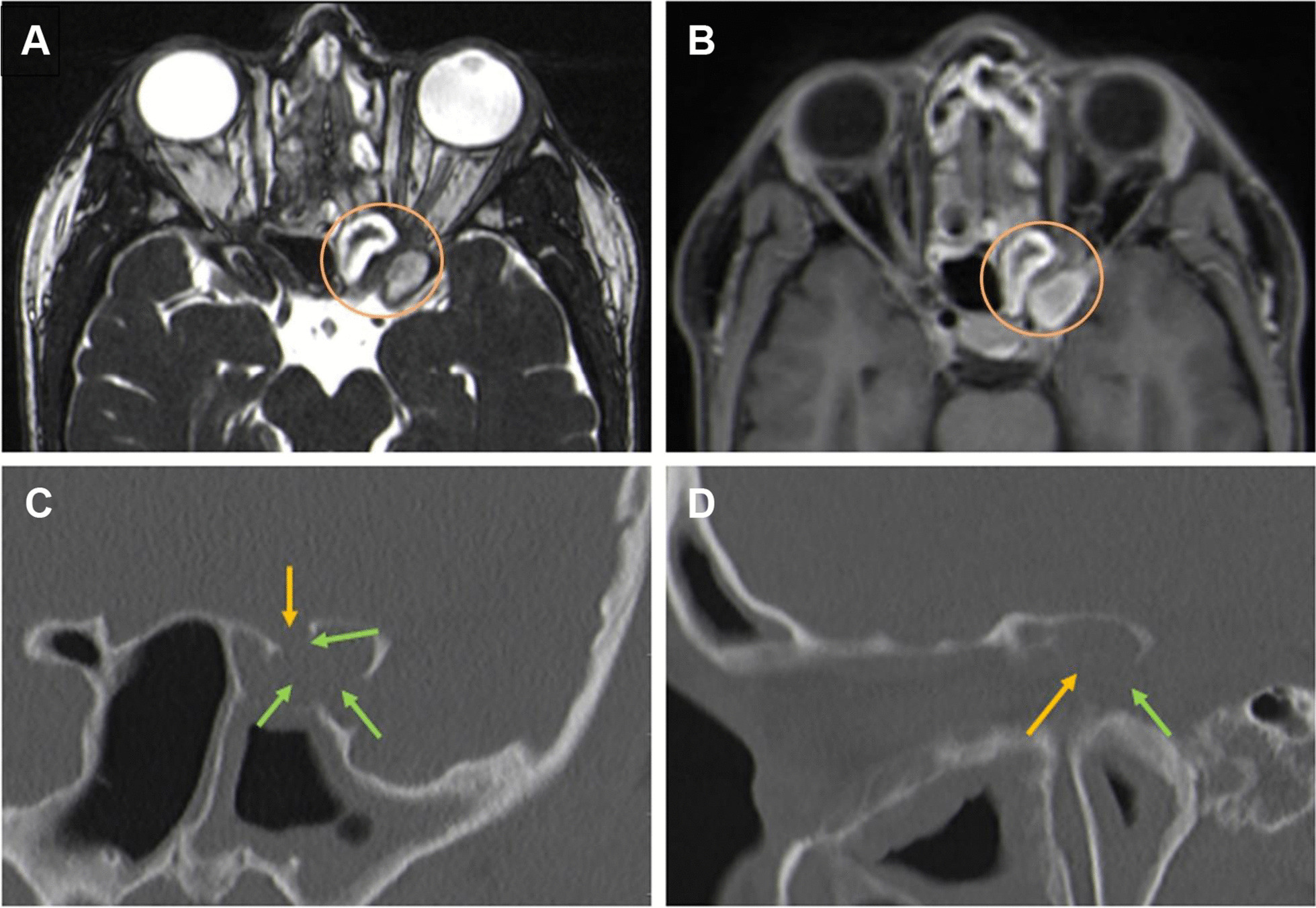
High-resolution cranial MRI and CT on admission. The high-resolution T2-weighted CISS images (**A**: axial plane) and contrast-enhanced T1-weighted SPACE FS (**B**: axial plane) show the course of the left optic nerve, with indentation and direct contact with the mucocele. No signs of optic nerve edema due to compression or ischemia are found (orange circle), supporting the additional presence of a local toxic impact. High-resolution CT (**C**: coronal plane; **D**: parasagittal plane) shows the shadowing of this Onodi cell, caused by a mucocele, as identified by the MRI sequences. Thinned bone lamellae or partial bone resorption (green arrows) and expanded bony margins are in direct contact with the course of the optic nerve (orange arrows)

Imaging revealed chronic rhinosinusitis, nasal polyps, and a left sphenoid sinus mucocele in direct contact with the left optic nerve (Fig. 1A, B). The absence of nerve compression or edema suggested optic neuropathy, necessitating urgent surgical decompression. A subsequent computed tomography (CT) scan revealed thinning or partial absence of bony structures in the parasphenoidal cell, likely owing to pressure atrophy from the expanding mucocele (Fig. 1C, D).

**Fig. 2:**
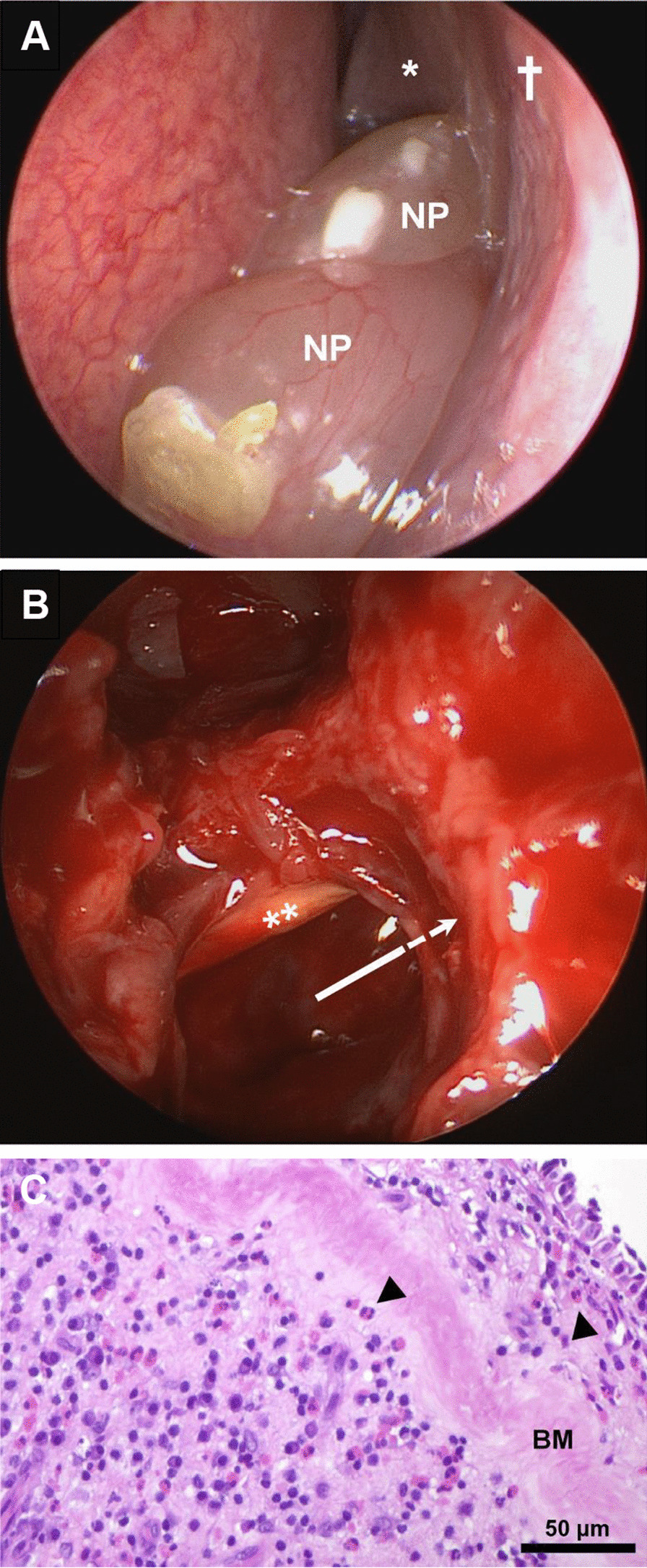
Endoscopic view of the left nasal cavity before sinus surgery (**A**) and directly afterward (**B**), and histopathological examination of the polyp removed from the left sphenoid sinus (**C**). The middle nasal concha (**A**, asterisk) and the orbital plate of the ethmoid bone (lamina papyracea; **A**, cross) surround the ethmoid sinus complex. Recurrent nasal polyps (**A**, NP) are congesting the nasal cavity and extending up to and within the left sphenoid sinus and the adjacent Onodi cell more posteriorly. During surgery, the remaining bony septa that had enclosed the left optic nerve were removed (**B**, double asterisk) and the sphenoid sinus and adjacent Onodi cell (**B**, arrow) were widely opened. Histopathological analysis of the polyp from the sphenoid sinus (**C**; hematoxylin–eosin staining), which had been in direct contact with the optic nerve, revealed thickened basement membranes (panel C, BM) and increased tissue eosinophilia (**C**, arrowheads), suggesting a type 2 inflammatory endotype

Emergency revision sinus surgery was conducted with decompression of the optic nerve (Fig. 2). Postsurgery treatment included methylprednisolone, ceftriaxone, vitamins [cyanocobalamin (vitamin B_12_), pyridoxine (vitamin B_6_) and thiamine (vitamin B_1_)], and continuation of existing medications. On the third day post surgery, intraocular pressure increased (48 mmHg bilaterally), suggesting a steroid response. Administration of methylprednisolone was halted, and the pressure was promptly reduced with acetazolamide, intraocular dorzolamide, and apraclonidine to 29 mmHg.

Histopathological analysis indicated chronic sinusitis, polyp presence, and elevated tissue eosinophilia, pointing to a type 2 inflammatory endotype consistent with an allergic or NERD etiopathogenesis. There was no evidence of malignancy or infection, aligning with the negative microbiological results.

**Fig. 3:**
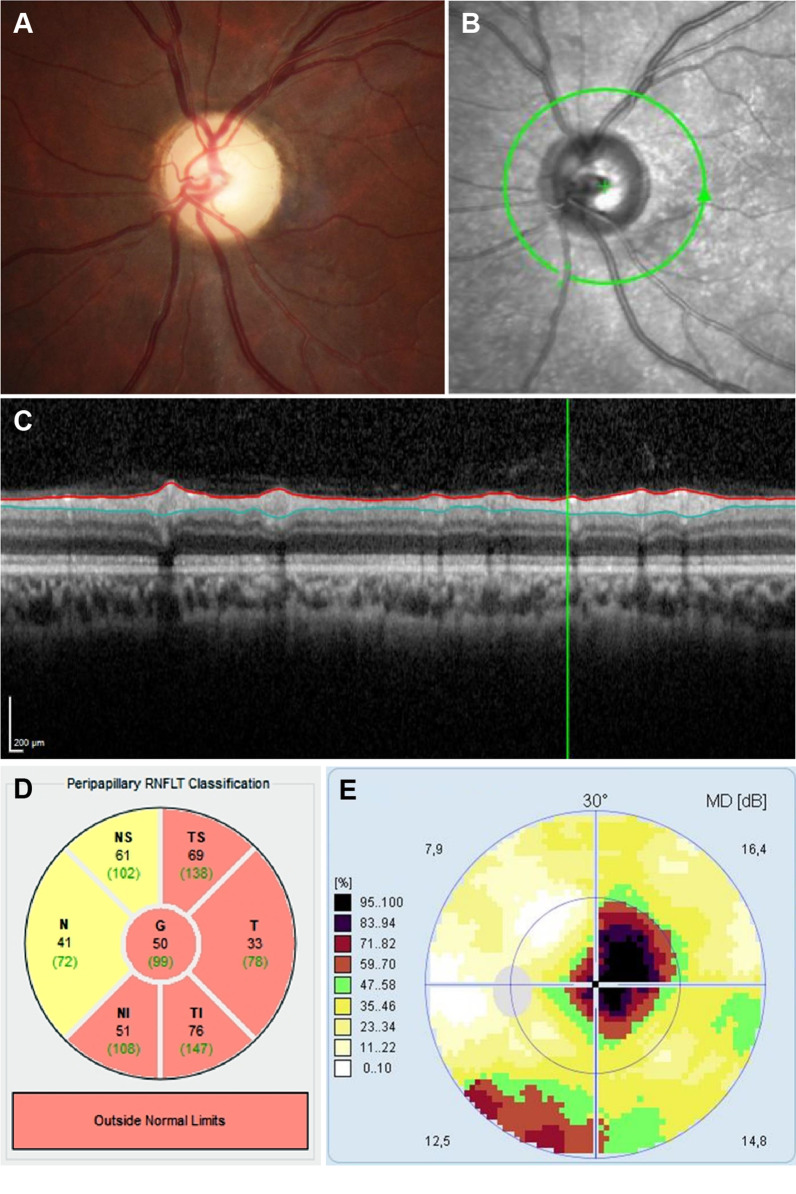
Ophthalmologic examination of the affected left eye, 3 months after treatment. A pale optic disk on fundus imaging (**A**) and a brightened disk on OCTA infrared imaging (**B**) both indicate simple optic atrophy. The OCTA B-scan reveals the significantly reduced thickness of the peripapillary retinal nerve fiber layer (**C**; distance between red and blue lines), with a mean value of 50 μm (**D**: G, total; NS, nasal superior; N, nasal; NI, nasal inferior; TS, temporal superior; T, temporal; TI, temporal inferior). The perimetry and visual field examination both show a central scotoma, as indicated by black or brown areas (**E**; colors represent percentage loss of vision)

Twelve weeks post discharge, the patient’s left-eye visual acuity improved to 20/50 (Snellen), intraocular pressures were normal, and the anterior segment examination was regular. Ophthalmoscopy (Fig. 3A) confirmed optic atrophy, while OCTA showed a significant reduction in the retinal nerve fiber layer thickness (Fig. 3C, D). Perimetric assessment revealed the presence of a central scotoma (Fig. 3E). Six months post discharge, there was no recurrence of nasal polyps, but ophthalmological findings remained unchanged.

## Discussion

The presented case deals with unilateral sudden vision loss, symptomatic of optic neuritis. Typically, optic neuritis manifests unilaterally, frequently in association with multiple sclerosis, resulting in progressive axonal damage and demyelination. Atypical optic neuritis, on the other hand, arises from diverse underlying conditions such as temporal arteritis, Devic syndrome, sarcoidosis, lupus erythematosus, chronic relapsing inflammatory optic neuropathy, and Leber’s hereditary optic neuropathy. Post-vaccine reactions and para- or postinfectious states (Lyme disease, syphilis, tuberculosis, cytomegalovirus infection, toxoplasmosis, *Bartonella* infection), tumor infiltration, metabolic deficiency, toxic damage, and neuronal ischemia also need to be considered.

Considering this patient’s unremarkable additional findings, normal OCTA results, and absence of significant clinical history, the possibility of autoimmune diseases, ischemia, or tumor infiltration was ruled out. The patient’s history of NERD, recurrent sinus surgery, and nonadherence to medication suggested the possibility of recurrent rhinosinusitis, which was confirmed through sinonasal endoscopy.

Given the dominance of vision loss in this patient’s symptomatology, magnetic resonance imaging (MRI) was selected as the appropriate imaging modality [3]. In conjunction with CT imaging—necessitated by the patient’s history—these techniques (Fig. 1) unveiled a cystic lesion in the left sphenoid sinus and thinned bone lamellae with partial bone resorption (Fig. 1E, F; green arrows), implying the need for immediate surgical intervention. Chronic rhinosinusitis (CRS), an inflammatory disease with significant prevalence [2, 4], is broadly classified into type 2 inflammation and non-type 2 inflammation categories [5]. Both CRS with nasal polyps (CRSwNP) and eosinophilic CRS represent type 2 inflammations, with about 10% of CRSwNP patients also suffering from NERD [6, 7]. In patients with NERD, an augmented leukotriene synthesis [8, 9] compounds the effects of type 2 inflammation, thereby exacerbating the chronic eosinophilic airway inflammation. As observed in this case, serum eosinophilia may also present as a supplementary finding.

The therapeutic approach for CRSwNP encompasses regular applications of intranasal glucocorticoids, large-volume nasal rinsing, and, for severe cases, oral corticoids and sinus surgery [8]. Additionally, acetylsalicylic acid intake [10] and dupilumab therapy [11–13] have shown promising results. It is noteworthy, however, that postsurgery recurrence of nasal polyps is common, especially in NERD patients, potentially necessitating revision sinus surgery [7, 14]. The use of high-dose methylprednisolone for acute optic neuritis, originally adapted from acute spinal cord trauma treatment recommendations, remains contentious, with follow-up studies failing to confirm substantial benefits [15]. However, considering the pros and cons, we opted for high-dose corticosteroid therapy in this instance. Rigorous monitoring is essential to promptly identify and manage potential side effects, such as the acute steroid glaucoma observed in this patient.

## Conclusion

Diagnosing and managing optic neuritis demands an interdisciplinary approach. In this patient’s case, her NERD history and increased tissue eosinophilia guided the diagnosis. Effective treatment of CRS entails consistent and early therapy to enhance patient quality of life and avert severe complications. Appreciation of type 2 inflammations and emerging therapies such as dupilumab is crucial. However, acute complications call for immediate surgical interventions to alleviate nerve trauma and toxicity, mitigating progressive or irreversible deficits.

## Data Availability

Not applicable.

## Abbreviations

ASA: Acetylsalicylic acid
CISS: Constructive interference in steady state
COX-1: Cyclooxygenase 1
CRS: Chronic rhinosinusitis
CRSsNP: Chronic rhinosinusitis without (sine) nasal polyps
CRSwNP: Chronic rhinosinusitis with nasal polyps
CSF: Cerebrospinal fluid
CT: Computed tomography
FS: Fat saturation
IL: Interleukin
MRI: Magnetic resonance imaging
NERD: Nonsteroidal antiinflammatory drug-exacerbated respiratory disease
NSAID: Nonsteroidal anti-inflammatory drug
OCTA: Optical coherence tomography angiography
SPACE: Sampling perfection with application-optimized contrast using different flip angle evolutions

## References

[1] Abel A, McClelland C, Lee MS (2019). Critical review: typical and atypical optic neuritis. Surv Ophthalmol.

[2] DeConde AS, Soler ZM (2016). Chronic rhinosinusitis: epidemiology and burden of disease. Am J Rhinol Allergy.

[3] Galletti B, Gazia F, Galletti C (2019). Endoscopic treatment of a periorbital fat herniation caused by spontaneous solution of continuity of the papyracea lamina. BMJ Case Rep.

[4] Fokkens WJ, Lund VJ, Mullol J (2012). European position paper on rhinosinusitis and nasal polyps 2012. Rhinol Suppl.

[5] Fokkens WJ, Lund VJ, Hopkins C (2020). European position paper on rhinosinusitis and nasal polyps 2020. Rhinology.

[6] Rajan JP, Wineinger NE, Stevenson DD (2015). Prevalence of aspirin-exacerbated respiratory disease among asthmatic patients: a meta-analysis of the literature. J Allergy Clin Immunol.

[7] Stevens WW, Peters AT, Hirsch AG (2017). Clinical characteristics of patients with chronic rhinosinusitis with nasal polyps, asthma, and aspirin-exacerbated respiratory disease. J Allergy Clin Immunol Pract.

[8] Cahill KN, Boyce JA (2017). Aspirin-exacerbated respiratory disease: mediators and mechanisms of a clinical disease. J Allergy Clin Immunol.

[9] Tan BK, Wang Y (2019). Do NERDy eosinophils accelerate nasal polyp growth?. Allergy.

[10] Larivee N, Chin CJ (2020). Aspirin desensitization therapy in aspirin-exacerbated respiratory disease: a systematic review. Int Forum Allergy Rhinol.

[11] Shirley M (2017). Dupilumab: first global approval. Drugs.

[12] Bachert C, Han JK, Desrosiers M (2019). Efficacy and safety of dupilumab in patients with severe chronic rhinosinusitis with nasal polyps (LIBERTY NP SINUS-24 and LIBERTY NP SINUS-52): results from two multicentre, randomised, double-blind, placebo-controlled, parallel-group phase 3 trials. Lancet.

[13] Mustafa SS, Vadamalai K, Scott B (2021). Dupilumab as add-on therapy for chronic rhinosinusitis with nasal polyposis in aspirin exacerbated respiratory disease. Am J Rhinol Allergy.

[14] Galletti B, Gazia F, Freni F (2019). Endoscopic sinus surgery with and without computer assisted navigation: a retrospective study. Auris Nasus Larynx.

[15] Nesathurai S (1998). Steroids and spinal cord injury: revisiting the NASCIS 2 and NASCIS 3 trials. J Trauma.

